# Reliability and validity of an Italian four-level emergency triage system

**DOI:** 10.1186/cc11089

**Published:** 2012-03-20

**Authors:** N Parenti, G Rastelli, C Ferri, V Serventi, R Lazzari, L Sarli

**Affiliations:** 1 University of Parma, Bologna, Italy; 2 Ospedale Fidenza, Italy; 3 University of Modena, Italy

## Introduction

The goal of this study is to assess the reliability and validity of a four-level emergency triage system (Urgency Category (UC) 1 = immediate response; UC 2, 3 and 4 assessment within 20, 60 and 120 minutes respectively) used in an Italian large urban hospital with 60,000 emergency department (ED) visits annually.

## Methods

Three triage nurses, using our triage system, independently assigned, at the same time, triage scores to each patient admitted to the ED from June to August 2011. We collected demographic and clinical characteristics, nurse triage category, resources used for each triage code (for example, laboratory tests, EKG, radiographs, procedures), admission status and site, nurse triage forms that included presenting complaint, vital signs, and pain score. For each scenario, the most frequent UC (the mode) has been considered as true triage. Weighted kappa (K) was used to calculate inter-rater reliability. Validity was evaluated by studying the relationships between the triage category assigned by the nurses and resource consumption.

## Results

A total of 315 patients admitted to the ED were included in the study randomly (35 were excluded for incomplete data). Mean age was 47 years. Five patients were admitted to the ICU, 48 to nonintensive units. Trauma was the most frequent symptom at triage (44%). The mean time of rating was 2 minutes. The UCs assigned were: 14% with UC 4, 60% UC 3, 25.7% UC 2, 0.3% UC 1. We found 2/315 (0.6%) cases with a marked discordance (2 or more points), 69/315 (21.9%) cases with partial agreement (2/3) and 244/315 (77.5%) cases with a complete agreement (5/5) among nurses who used the triage method. Interrater reliability among the three nurses was K = 0.71 (CI: 0.58 to 0.84). Hospital admission by our triage system was as follows: 1 (100%), 2 (30%), 3 (12%), 4 (2%). The mean of resources used for each triage code was: 4.5 (SD 2.2) for UC 2; 3.2 (SD = 1.67) for UC 3; 1.89 (SD 0.84) for UC 4.

## Conclusion

Our triage system shows a good interrater reliability and validity in predicting resource consumption. To our knowledge, this is the first prospective Italian study that tests the relationships between the triage category assigned by the nurses (using a four-level triage method) and resource consumption.

